# The estimation of non-irrigated crop area and production using the regression analysis approach: A case study of Bursa Region (Turkey) in the mid-nineteenth century

**DOI:** 10.1371/journal.pone.0251091

**Published:** 2021-04-30

**Authors:** Eda Ustaoglu, M. Erdem Kabadayı, Petrus Johannes Gerrits

**Affiliations:** College of Social Sciences and Humanities, Koç University, Sarıyer/Istanbul, Turkey; United Nations University Institute for Natural Resources in Africa, GHANA

## Abstract

Agricultural land cover and its changing extent are directly related to human activities, which have an adverse impact on the environment and ecosystems. The historical knowledge of crop production and its cultivation area is a key element. Such data provide a base for monitoring and mapping spatio-temporal changes in agricultural land cover/use, which is of great significance to examine its impacts on environmental systems. Historical maps and related data obtained from historical archives can be effectively used for reconstruction purposes through using sample data from ground observations, government inventories, or other historical sources. This study considered historical population and cropland survey data obtained from Ottoman Archives and cropland suitability map, accessibility, and geophysical attributes as ancillary data to estimate non-irrigated crop production and its corresponding cultivation area in the 1840s Bursa Region, Turkey. We used the regression analysis approach to estimate agricultural land area and grain production for the unknown data points in the study region. We provide the spatial distribution of production and its cultivation area based on the estimates of regression models. The reconstruction can be used in line with future historical research aiming to model landscape, climate, and ecosystems to assess the impact of human activities on the environmental systems in preindustrial times in the Bursa Region context.

## 1. Introduction

The terrestrial biosphere has been continually changing to meet demands for food, feed, fiber, fuel, and habitation [1, 2]. The direct human-induced impacts on land use have been the conversion of natural landscapes to agriculture [3]. The literature has argued that the most productive land area is becoming scarce, and this situation will shape future agricultural production [4–6]. The natural environment changes have influenced many critical biogeochemical cycles such as carbon, phosphorus, and nitrogen cycles [7]. These changes have resulted in increased levels of greenhouse gases [8], essential implications on the health of aquatic ecosystems [9], land surface energy imbalances [10] and has altered Earth’s climate [11]. Therefore, many efforts have been put forward to analyze and quantify land cover/use dynamics to assess its impacts on the natural environment and ecosystems [12].

The primary driver of agricultural land expansion is the growing human population. From 1800 to 2000, the world population has risen about six-fold from less than one billion to six billion [13]. Agricultural production in the same period has increased relatively faster, i.e., more than tenfold [14]. Conversion of the natural landscape to agriculture can be considered the most significant factor having the greatest impact on the environment. Over the past 300 years, estimates for the decrease in forestland due to agricultural expansion range from 8 to 13 million km^2^ [15]. Conversion to agriculture has resulted in increased surface runoff, soil erosion, land degradation, loss of biodiversity and ecosystem services, and adverse impacts on climate systems [16, 17]. Other land cover changes such as establishing permanent grassland/pastures or afforestation could increase carbon storage in the soil and reduce greenhouse gases and preserve the environment [18].

Agricultural production is a function of the land area under cultivation and the intensity of cropping on the cultivated land. Therefore, the volume of agricultural output is linked to changes in the total area under cultivation and changes in cropping intensity [19, 20]. An increase in agricultural land area is named agricultural expansion, whereas a decline in cultivated land is denoted as contraction. A decrease in land use and contraction intensity can be due to farmland abandonment [21] as well as the conversion of agricultural land to other land uses such as urban land use [22, 23]. Intensification and extensification are the processes of increasing and decreasing the use of capital and inputs (e.g. fertilizers, pesticides, labor, and machinery) relative to the land area [24]. Production increases are directly linked to agricultural expansion or intensification of cropland, which depend on the amount of land available and suitable for cultivation [19, 20]. To assess the environmental impacts of agricultural land expansion and intensification of production, information is needed on the current and historical patterns of agricultural and other land use activities.

It is essential to use quantitative information in the study of land cover/use pattern and cropland change analysis to examine the environmental impacts of such changes [25]. Numerical and spatially explicit models regarding historic land cover/use and cropland cover are increasingly being developed at different levels. At the global level, two significant studies can be highlighted. The Center for Sustainability and the Global Environment (SAGE) created a global cropland dataset for 1700 and 1992 through adopting contemporary and historical inventories of cropland and a ’hindcast modeling’ method [26]. The Netherlands Environmental Assessment Agency, developed another global historical dataset, the History Database of the Global Environment (HYDE) [27]. The most recent HYDE 3.1 dataset developed a spatially gridded reconstruction of cropland covering the past 12,000 year period. There are also other global historical reconstructions based on various data and modeling techniques, including Houghton [28], Hurtt et al. [29], and Pongratz et al. [30]. At the continental level, Fuchs et al. [31] reconstructed spatially explicit historical land change data for Europe using historical aerial photographs from 1950 to 1990. Kaplan et al. [32] is another example at the European scale, which developed a dataset for forest cover over the last three millennia (see also [33]). The reconstructions of agricultural land were also undertaken at the country level [12, 25, 34, 35] while others are at the more regional or provincial level [36–38].

These studies, in common, integrated remotely sensed data on the contemporary patterns of agriculture with historical data on agriculture and population to develop spatially explicit reconstructions of land cover/use (e.g. crops, pastures, forest cover) over the past centuries or the past decades. Though these datasets provide spatially and temporally continuous gridded data on agricultural land cover/use, they have coarse spatial resolution, which reduces their application potential in local study areas. Some argued that these data have been applied in a general context to examine historical cropland change patterns; therefore, they should only be used for country-to-global scale analysis and modeling [25, 39, 40].

Local-scale analysis has contributed to the historical cropland reconstructions, given that local datasets can help verify global and regional scale datasets and models. Some examples of land use reconstructions based on detailed real data (e.g. historical maps or documents) for the local case study areas or smaller regions are Cousins et al. [41], Benitez and Fisher [42], Skalos et al. [43], Godet and Thomas [44], Kaim et al. [45], Loran et al. [46], Brandolini et al. [47]. Such studies are essential in land change analysis, given that these studies provide fine-scale spatial and temporal data on the historical land use patterns and change. However, significant challenges exist to apply these methods in the Turkish context, given that the data required to provide historical estimates are scarce. Turkish literature covers studies of landscape reconstruction, particularly in the prehistoric periods, for instance, for Konya Basin in south-central Anatolia [48]; the ancient city of the Sagalassos in western Taurus mountain range [49]; northern Mesopotamia and central Anatolia [50]; Roman agricultural practices in central Turkey [51]; Çatalhöyük in central-southern Turkey [52] and Burdur Province in south-west Anatolia [53]. Despite the growing literature on agriculture and land use systems in the prehistoric periods, there is hardly any study on agricultural land reconstructions conducted for Turkey’s recent historical periods.

Based on the methods employed to reconstruct historic land cover/use, this study aims to create a new dataset of total area of cultivation and total production volume for grains in our selected case study area, i.e. Bursa Region in Turkey in the 1840s. Bursa Region is located in the north-western part of Turkey, which has been considered as one of the most significant settlements starting from the Ottoman Era till modern times. The economic history of the late Ottoman Empire and/or the Turkish Republic has not used region as a unit of analysis adequately. Therefore, we severely lack empirically grounded historical studies on regions’ economic performances of today’s Turkey. The most recent and robust historical national accounts [54] are also on national level and unlike the recent developments on focusing on regional historical GDP estimates of selected European countries [55], there are almost no regional historical GDP estimates for Turkey. For the urban and industrial core of the region, the city of Bursa, on the other hand, there is a well-developed literature on its economic history for the nineteenth century [56–58]. Bursa was a regional industrial center and a hub for the Ottoman Empire’s silk production [59, 60]. However, for the region’s economic evaluation in the nineteenth century, we have no encompassing studies and have to rely on contemporary accounts mainly based on European observers and consular reports [61]. These contemporary sources highlight export-oriented agro-industrial products such as mulberry groves for silk production, olive, and grape production, yet no surplus but subsistence level of grain and/or rice production for the region. Based on historical population and cropland survey data obtained from Ottoman Archives and using cropland suitability map, accessibility, and geophysical attributes as ancillary data, we estimated cropland area and the amount of production of non-irrigated crops, which consisted of mainly grains in the 1840s for around 576 settlements in Bursa Region using the regression analysis techniques.

## 2. Modeling agricultural land market dynamics

Historically, the most significant factor influencing agricultural product demand has been population growth [62, 63]. Ramankutty et al. [64] analyzed the population against hectares of cropland in 1900. They found a positive correlation between population and cropland areas, with the global average cropland area equal to 0.76 ha per capita. Coming to the end of the 1900s, the authors noted that the positive correlation persists but with a reduction of global average cropland area to 0.35 ha per capita [64]. In modern industrial economies, the global population is the main controlling factor in the amount of global agricultural land. At the same time, in the preindustrial periods, it had been a local or regional population that controlled the local extent of agriculture [65, 66]. Since we focus on preindustrial times in the current study, we admit that the increase in the local/regional population led to an increase in the total amount of agricultural production and land area in our case study region. A further issue is the population pressure and land scarcity that may lead to an intensification of the agricultural land, driven by increased demands for land-based products and services [67]. Land-use intensification process is associated with technological innovations to raise agricultural yield from the given amount of land [22]. Regarding majority of settlements in our case study, population and food demand are considerably lower than the amount of available land suitable for agricultural production. Therefore we presume that to a large extent there is no land intensification observed in the Bursa Region in the 1840s.

The income level of societies is another factor that is positively associated with agricultural land take. As income increases, households’ demand for goods, including food, increases, which require greater agricultural area to sustain the increasing demand of wealthy societies [68, 69]. Food consumption patterns may have a significant influence on the land requirements for food. For instance, increasing indirect grain consumption (i.e. animal products) rather than direct grain consumption results in increasing per capita land requirements [70, 71]. The indirect grain consumption is induced by economic growth and changing patterns of food consumption. Due to data availability issues, the land required for animal products is not included in the study, but only the land requirements for grain consumption (e.g. non-irrigated crops) were considered. Other factors may affect the agricultural land demand, such as socioeconomic factors (e.g. input and output prices; farm income, household size, age, education level of farmers), water availability, and policy influences such as taxes and subsidies. Technological improvements in agricultural production are another factor explaining the agricultural demand, given that advances in agricultural production will result in relatively more minor increases in agricultural lands. Concerning the Bursa Region, advancements in agricultural technologies were not explored in the study since traditional agricultural techniques, rather than technological improvements, were predominantly applied in the Region in the nineteenth century.

In Chen et al.’s [72] explanation, the main factors influencing the grain demand are population size, per capita grain demand, and self-sufficiency ratio. The authors claim that rural people’s grain consumption is much higher than that of urban people as the latter more depend on indirect grain consumption [72, 73]. According to Lu [74], grain demand per capita is 400 kg of grain per year of subsistence and 400–600 kg for a moderately prosperous life. While Chen [75] predicts grain demand per capita at moderately prosperous and prosperous levels is 450 kg and 500 kg per year. We do not have significant variations on the per capita demand for grain intake in the Bursa Region. The region’s settlements are homogeneously distributed, having a predominantly rural population with less apparent urban-rural differences. The grain self-sufficiency ratio represents the ratio of grain produced by a country or region to the grain demand of country, region, or local area. The self-sufficiency ratio of more than 1 indicates total self-sufficiency; between 0.9 and 1 means low-to-high self-sufficiency, and less than 0.9 means a high risk of food security [72]. In our case study, nearly all of the settlements in Bursa Region were self-sufficient. Many of them were not importing agricultural products from other settlements/regions, neither were they exporting their products to other locations. Therefore, the self-sufficiency ratio is assumed to equal to 1. Based on these explanations, the general form of agricultural land demand can be specified as

$$D_{L,i,t}=F\left(X_{i,t}\right)+v_{i,t}$$ (1)

where D_L,i,t_ is the agricultural land demand for settlement i at time t; X_i,t_ is a matrix of explanatory variables including socio-economic factors (e.g. farm income, population, number of households), location, accessibility and geo-physical variables, institutional/policy variables, and others; and v_i,t_ is the random disturbance term. The land supply function is provided in Eq (2) where agricultural land supply is related to land restrictions such as land zoning or natural limitations such as protected areas, water bodies or land unsuitable for agriculture (Y_i,t_); and random disturbance term (u_i,t_).

$$S_{L,i,t}=F\left(Y_{i,t}\right)+u_{i,t}$$ (2)

Solving the equilibrium of demand (Eq 1) and supply (Eq 2), a reduced form of the new equation, AGRI_LAND_i,t_, explicating the conditions of demand and supply in the agricultural land market can be formed as:

$$AGRI\_LAND_{i,t}=F\left(X_{i,t},Y_{i,t}\right)+e_{i,t}$$ (3)

Because land rent data is usually unavailable as it is in our case, it is common in the literature to approximate them using other variables [18]. Therefore, to represent the rental prices in the 1840s, we used location, accessibility, and land quality as proxies for agricultural land’s rental price. Regarding the supply function, we included natural restrictions, including the area of water bodies and land unsuitable for agriculture in the regressions as an explanatory variable, but its coefficient was estimated insignificant. The reason can be that there is an abundance of land suitable for agriculture in the Bursa Region. Therefore the supply restrictions have only a minor impact on the developed land for agriculture. The land abundance in the Bursa Region was also verified by MacFarlane [61, p.370], asserting that "*…In the country above the plain they get a crop of wheat off a field and then leave it fallow for a year or two*, *saying that they have so much ground they need not over-fatigue it…"*. Spatial data on land zonings or protected land in the Bursa Region is not available for the 1840s; therefore, these variables were discarded from the analysis.

## 3. Data and methodology

### 3.1. Data

Our data source is an empire-wide tax survey from 1845 (*temettuat* is the original Ottoman/Turkish name of the survey) which represents data at one point in time [76]. This survey has covered a substantial area of the Ottoman Empire with some exceptions and provides very detailed information on all income-generating assets of all households in the surveyed locations and therefore is a combination of industrial, occupational, and most importantly for our purposes, agricultural censuses. This survey has unique data coverage per household and was never surpassed before or after its completion in 1845. *Temettuat* covers a wide variety of variables. This survey results from a tax reform aiming to register all income yielding assets per household, mainly under the headings of agricultural production, animal husbandry, occupational revenues, and rented property. In rural settings such as the Bursa Region under consideration, the survey has registered detailed agrarian data. The most important two are the cultivation area in units convertible to hectares and the volume of products to be taxed in kind in units convertible to tons. For this study, we extracted these two variables. One of the challenges to work with this survey is the impossibility of assessing its reliability. The data conveyed with the *temettuat* is unprecedented in detail, yet it provides only one single data point. The agricultural data it provides cannot be double-checked by any other contemporary or later source. It is assumed that it covers around one million households in the Ottoman Empire’s core regions in Southeast Europe and Anatolia. Approximately 18,000 registers of this survey are available in the Ottoman state archives since the 1990s, yet only on the very micro level handwritten data format as it was collected by the clerks on the field in the mid-nineteenth century. The survey’s results were never tabulated or subject to any systematic inquiry during the Ottoman era. Since the 1990s, there was no organized effort to utilize them in economic history either. These registers hitherto have been used almost exclusively in geographically limited case studies dedicated to individual cities, towns, or sets of villages. There are very few exceptions to the underutilization of this rich source.

Recently, based upon teamwork of source digitization, a total of around 800 *temettuat* registers were curated from sixteen urban locations to compare occupational structures and ethno-religious affiliations of more than 50,000 Ottoman subjects [77]. However, the survey’s untapped potential lies in utilizing the data it conveys on product type-specific agricultural production both in areas of cultivated land and total produce in rural settings. These data are suitable to aggregate for the entire Bursa Region, which corresponds to the NUTS 3 (Nomenclature of Terrestrial Units for Statistics) level for Turkey. Estimating crop yields and total acreages designated to staples and their total production volumes for the 1840s would serve as a base year for long-term historical examinations and enable future studies going to the beginning of the commercialization of agriculture and incorporation of Ottoman economy to the world structures. Our unit of analysis in this exercise is a regional one. To the best of our knowledge, only two studies used this survey for regional agricultural production comparisons, yet both with limitations. Koyuncu and Küçükkalay [78] sampled a total of 20 villages to compare three regions’ economic structures regarding specialization in occupations and distribution of income, wealth, and taxation in the 1840s. Nevertheless, Koyuncu and Küçükkalay [78] sampled their villages without devising a sampling strategy. Therefore, their selected villages’ representativeness, especially as few as five in two regions in Anatolia, is not very convincing. The other study is based upon a geo-sampling strategy; however, it estimates only the shares of grain types in agricultural mixes as proxies for export orientation—neither total area of cultivation nor production volume for grains in two regions [79].

In this study, we use a more comprehensive geo-sampling method to estimate total areas and total volume of grain cultivation for all 576 settlements to cover the entire Bursa Region except the city of Bursa. Our sampled dataset has the entire households in 72 settlements. We extracted the total area of cultivation devoted to grains and the total volume of grain production of these 72 settlements from the survey manually. Initially, our sample had 88 settlements. The data curation and coding procedures for both crop area and crop yields 16 settlements did not qualify for our analysis. The initial 88 settlements had in total 5266 households, with 17,126 agricultural production area and 18,704 agricultural production volume entries. Our data entry team spent approximately 130 workdays (40 households per workday) to read, extract and enter this information from handwritten Ottoman script archival sources into our MS Access relational database by using custom-made data entry tools. In the remaining 489 settlements, if we leave aside, the city of Bursa should have around 25,000 households. For a separate study, we already digitized and acquired data of 7,125 households registered in the city of Bursa. To extract necessary information on agricultural area and volume of production for the remaining settlements in the region, we would hypothetically need 625 workdays for the data entry. It would not be possible to conduct this total data entry since lack of complete sources in the archives. Therefore, with our estimations, we are modeling a total area and volume of crop production for a region, which cannot be reached via conventional historical methods. Covering the region totally ensures commensurability for longitudinal studies in agriculture. Ottoman statistical yearbooks from the late nineteenth and the first agriculture censuses from the early twentieth century and all successive agricultural censuses from the 1920s, 30s, and 40s of the Turkish Republic provide data on acreage and volume of production for grains for all sub-districts of Bursa. We want to make two explanatory notes on our methodology in making use of the 1840s survey. Firstly, there is a complication or a disconnect of data collection regarding agricultural production. The survey enumerates the total area of cultivation in Ottoman *dönüm*s, a unit of measurement easily convertible to hectares, for each production unit listed in types of cultivated land such as fields, vegetable gardens, orchards, or olive groves; without specifying the type or volume of production. In other words, the area of cultivation is registered more in terms of land use category and as area. The volumes of taxable agricultural production, encompassing all types of grain production, on the other hand, are listed in a different category in Ottoman *kile*, a unit of measurement easily convertible to tonnes without specifying the area of cultivation. We had to harmonize these two categories of data entry, land use, and agricultural production types, using one joint coding scheme to extract the total area and grain cultivation volume.

We opted for the Corine Land Cover (CLC) nomenclature of the European Union’s Earth Observation Programme (Copernicus). Since its initialization in 1985, the CLC inventory has been updated regularly, and we used the revised and supplemented nomenclature guidelines dated 10.05.2019 issued by the European Environment Agency [80]. Although CLC is a modern nomenclature for land cover and not for land use, it is highly compatible with the 1840s Ottoman survey. To our knowledge, CLC was not used in any historical studies. We coded all micro-level cultivated land entries belonging to individual households to the CLC in its highest detail level. Without any exception, we could code all cultivated land entries into the third level of detail in CLC into sub-categories, which would mean into further sub-categories of secondary level: 2.1 Arable land, 2.2 Permanent crops, 2.3 Pastures, and 2.4 Heterogeneous agricultural areas; of the first level category: 2. Agricultural areas. Among the third level sub-categories, 2.1.1 Non-irrigated arable land is the category with which we could code all survey area data devoted to grain production. We opted for CLC to code our survey data because of its dual suitability to code the agricultural tax data. The survey gives exact quantities of the main tithe tax on agricultural production. The tithe is a 10 percent in-kind tax levied mainly on grain production. Since all grain produce could also be coded into the same CLC category, 2.1.1 Non-irrigated arable land, we could aggregate the total area of cultivation in hectares and total volume of production of grains in tonnes per location for our sampled locations. We could estimate the totals in these two magnitudes for the entire region, enabling us to reach crop yield estimates for the whole region and spatial aggregation of the same projections for all sub-districts. Pre-census historical estimates of crop yields or agricultural productivity of land for the Ottoman Empire is a disputed topic. Orbay [81] recently provided a detailed review of structural difficulties, if not impossibilities, to estimate crop yields using financial sources in monetary values. Our approach of matching areas of cultivation with volumes of production coming from tithe in kind circumvents these difficulties.

We finally summarise statistics of the variables considered in the study categorized under six sub-titles (Table 1). These include land area and production, physical factors, accessibility, distance to water sources, soil quality, and socioeconomic factors. Land area and production were obtained from the 1845 *temettuat* survey. The total population and household numbers of the settlements were obtained from the contemporary population registers from the 1840s, again available in the Ottoman Archives. Distance to main residential centers and settlement centers were computed from the geocoded spatial data, which represented the central points of main residential centers (these were identified as the settlements assigned with the highest population numbers in the Bursa Region) and 576 settlements of Bursa. Distance to water sources and soil quality were developed from the spatial data showing the natural land cover and soil capabilities of the Bursa Region. The road network accessibility was computed using the 1940’s transportation network map, and finally, the elevation is from the Digital Elevation Model (DEM) dataset (Table 2). We acknowledge that these are the only available data that can be used to reconstruct grain production and corresponding land area in the nineteenth-century Bursa Region.

**Table 1:** Descriptive statistics of the variables.

Variable name (ABBREVIATION)	Mean	SD	Max	Min
***Land area and production***				
Land area of the non-irrigated crops (ha) (AGRI_LAND)	112.2	328.3	3020.0	0.18
Quantity of cultivated non-irrigated crops (tonne) (AGRI_PRODUCT)	75.9	1,000.0	582.2	0.2
***Physical factors***				
Average elevation (m) (ELEVATION)	384.2	256.1	966.7	12.3
**Accessibility**				
Average distance to roads (km) (DIST_ROADS)	0.55	0.31	1.80	0.21
Average distance to main residential centers (km) (DIST_RES_CENT)	14.72	13.25	61.80	2.81
Average distance to settlement centres (km) (DIST_SET_CENT)	1.90	0.58	4.10	1.03
***Distance to water sources***				
Average distance to water bodies (km) (DIST_WATER)	9.20	7.63	32.37	1.36
***Soil quality***				
Average of Agricultural Suitability Index (AGRI_SUIT)	5.49	1.17	8.19	3.58
***Socioeconomic factors***				
Total population of each settlement (POPULATION)	258.6	186.6	930.0	28.0
Total household number of each settlement (HH_NUM)	57.3	44.2	7	232

**Table 2:** Sources of data used in the analysis.

Data	Source	Year of Source Data
Elevation	DEM (SRTM, Shuttle Radar Topography Mission, NASA)	2000
Road network	Deutsche Heereskarte, Türkei	1943
Soil quality and land cover	Soil capability map	1970s
Residential and settlement centers	Geocoded settlement centers extracted from Ottoman population registers (NFS.d.), the Ottoman state archives	1840s
Population/Household number	Ottoman population registers	1840s
Data on area and volume of grain production	Ottoman survey *(temettuat*) (ML.VRD.TMT.d), the Ottoman State Archives	1840s

### 3.2. Agricultural land suitability assessment and sample selection methodology

We opted for a geo-sampling method instead of a purely random selection to better accommodate geographically varying factors determining grain cultivation in the region. Our model is based upon geolocated settlement center points. Clearly, the point data per settlement are not well-suited to sample possible territory on which grain produced in the settlements for the entire region. To geo-spatially estimate and to polygonize sampling areas, where possible fields assigned for grain production were, we used a rare collection of contemporary cadaster maps from Ottoman Archives: Presidency State Archives of the Republic of Turkey–Department of Ottoman Archives, HRT.h [82]. These maps from the 1850s cover seven rural settlements in the Bursa region with individual houses and fields in detail with a scale of 1:2000. To our knowledge, they have not been used in any study except Kabadayı et al. [79] again for similar geo-sampling purposes for a different part of the Empire. After georeferencing the cadaster maps and geolocating the farthest lying field suitable for grain production from a settlement central point, we calculated the time distance between the settlement mid-point and that field as 90 minutes using path distance geo-processing algorithm in ArcGIS for Desktop software, together with the commonly used Tobler hiking function for converting distance into time and energy expense. Then we applied this maximum walking distance result as the outer boundary for all settlements to create polygons for broad territory for grain cultivation.

**Fig 1:**
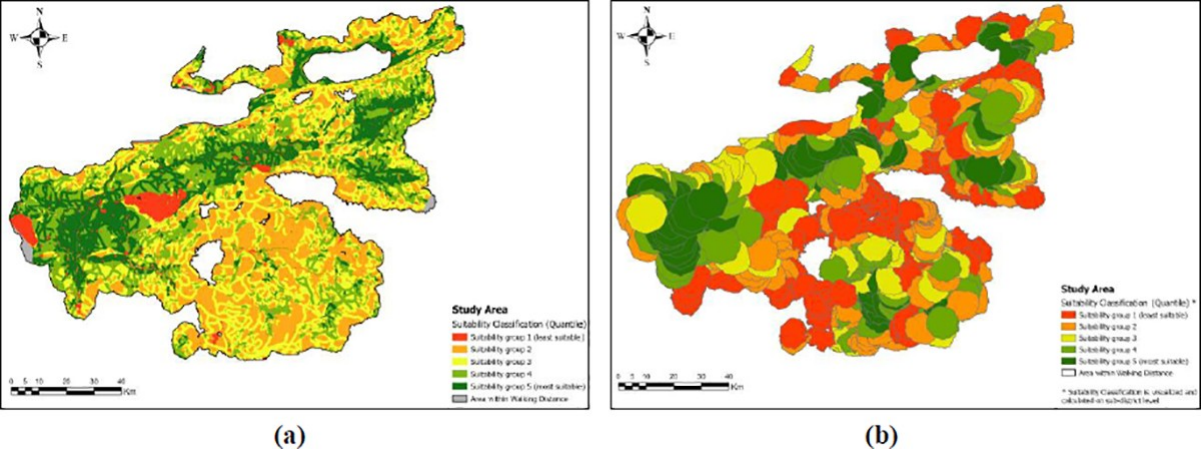
Suitability maps for agricultural development (warm colors represent the least suitability classes whereas cool colors represent the highest suitability).

After setting the boundaries for all settlements for the possible area of cultivation, we devised the suitability model. An optimal suitability model should accommodate socio-cultural, economic, topographic, and environmental criteria [83]. In our study, the suitability groups per sub-district are based on agricultural suitability of land for grain cultivation on the one hand and an estimate of available land per settlement on the other. The suitability groups were created using a suitability raster composed of land use capability classes (LCC) variables consisting of soil quality and quantity and ruggedness. Additionally, we include connectivity as a proxy of economic and demographic development into our suitability model. Connectivity in the region was measured by making use of a 1940s historical transport map [84]. Detailed reports on the condition of roads and agricultural transport facilities in the 1940s of Turkey manifest that the road infrastructure was severely underdeveloped [85]. We argue that the 1943 map is representative of the 1840s, especially for the rural transport network of the region. We used a weighted binary classification to pinpoint settlements within a 500-meter radius of a historical road and increased their overall connectedness in the region. The suitability raster and connectivity analysis were combined using a basic Analytical Hierarchical Process (AHP) and evaluated with a weight of 85% and 15%, respectively (Table 3) (Fig 1A). We assigned the dominant weight to natural endowments such as soil quality, depth, slope, elevation, and lesser weight to the transport infrastructure, exogenous to agricultural suitability.

**Table 3:** Composition of geo-sampling method layers.

Agricultural Productivity Assessment	Categories	Weight criteria (AHP)
*Suitability Raster (85%)*	I	9
	II	8.5
	III	8
	IV	7.5
	V	5
	VI	4.5
	VII	4
	VIII	1
*Connectivity (15%)*	< 500m	9
	> 500m	1

**Note**: suitability raster = land capability classes, connectivity = historical transport network, weighted criteria = 9 (very suitable) - 1. (least suitable).

**Fig 2:**
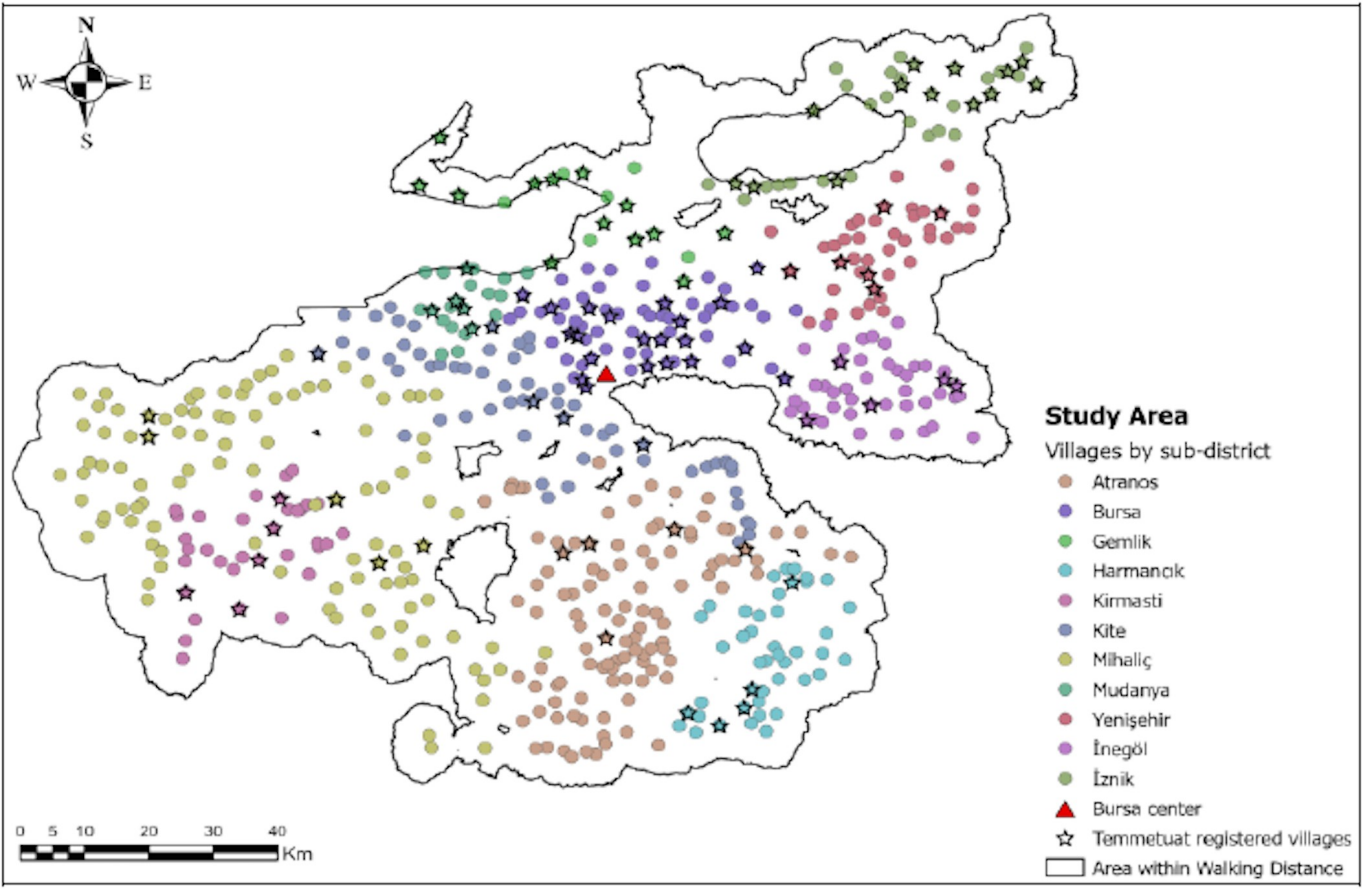
The study area.

Then we ranked all settlements into suitability groups within sub-districts in our study area, ranging from 1 to 5 (where the highest is the most suitable). We used a random sampling script with multiple constraints to pick at least one settlement from each suitability group per sub-district based on these classifications. This selection of at least five settlements also had to correspond to at least 10% of that sub-district’s total population and at the same time to 10% of the total number of households within that sub-district. Finally, we determined a weighted suitability index value per settlement by dividing the sum of total suitability by the associated territory and categorized them into respective suitability classes (Fig 1B), and extracted necessary information for chosen settlements’ *temettuat* registers obtained from the archives [82] (Presidency State Archives of the Republic of Turkey–Department of Ottoman Archives, ML.VRD.TMT.d.). The sub-districts and settlements and the location of the Bursa Region are presented in Fig 2.

### 3.3. Estimation methodology

This study aims to estimate the quantity of non-irrigated crops, especially grains, and their corresponding cultivation areas in the 1840s in the Bursa Region, Turkey. We applied regression analysis to both nonlinear and linear models for comparison purposes and selecting the best-fitted models. We developed nonlinear models estimated by Nonlinear Seemingly Unrelated Regression (NSUR) and Nonlinear Least Squares Regression (NLS) techniques and a linear OLS model to analyze the relationship between agricultural production and explanatory variables. Because agricultural production and the size of the agricultural land are correlated, we applied Zellner’s [86] well-known Seemingly Unrelated Regression (SUR) method to the system of nonlinear equations (i.e. NSUR) (see also [87]). For comparison purposes, we estimated the equations specified in the NSUR method separately by using the NLS method. The NLS method for estimation of the unknown parameters in the nonlinear function is conceptually the same as in the linear least squares regression (OLS). For the details of NLS, we refer to [88, 89].

**Fig 3:**
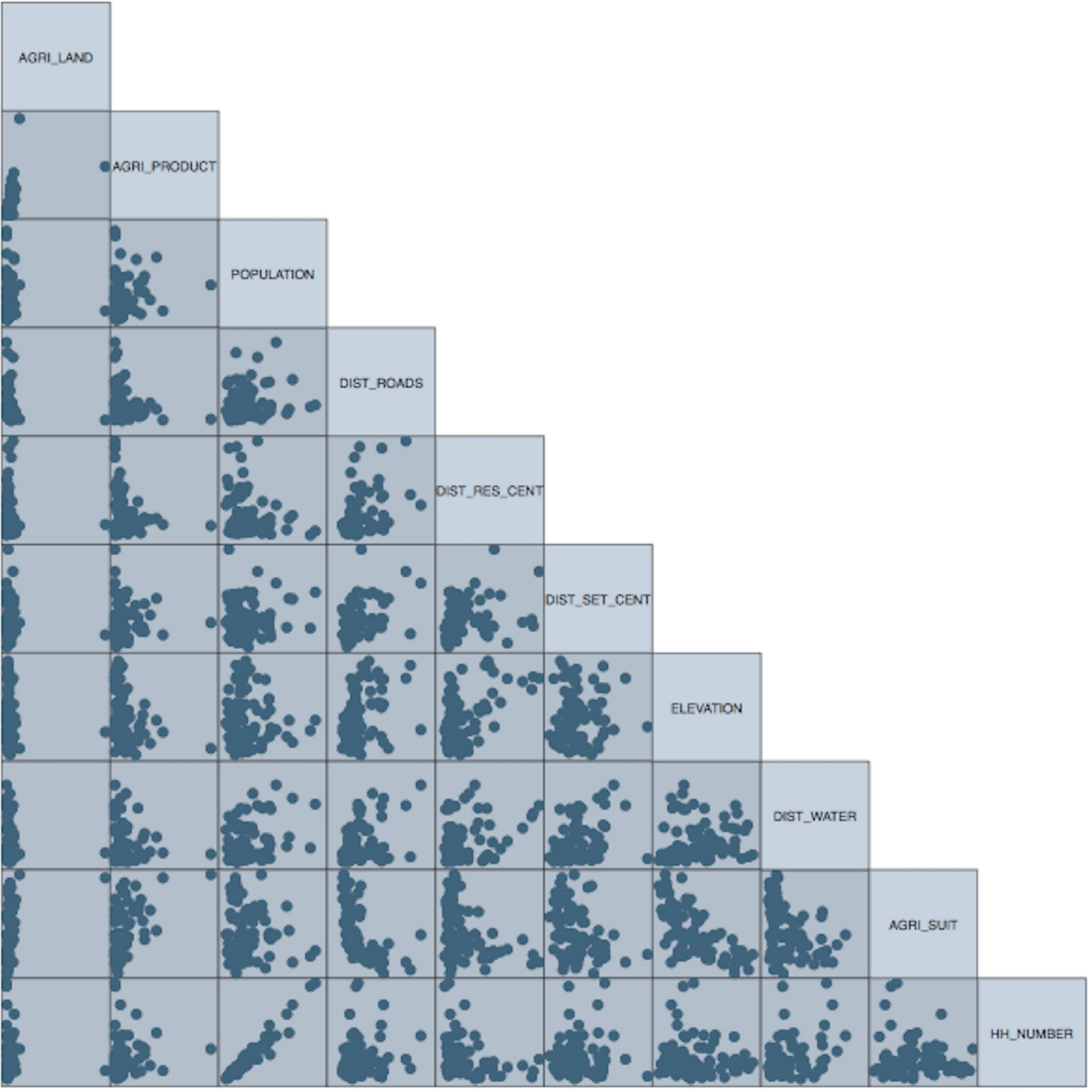
Scatter plot matrix showing the relationships between the variables (for the explanation of variables, please refer to Table 1).

Regarding the agricultural land area estimations, we only focus on linear models, including Truncated Poisson Regression (TPR), Quantile Regression (QREG), and Generalised Least Squares (GLS) methods. The reason for using the TPR model was to prevent zero and negative agricultural land area estimations. The TPR model details can be found in Grogger and Carson [90]; Long Scott [91] and Long Scott and Freese [92]. The QREG and GLS models were used to deal with heterogeneous variances, which resulted in a right-skewed distribution of observations of agricultural land area data. This is the case in our regression models, particularly the model with the agricultural land area being the response variable, which has a right-skewed distribution. This can be observed from Fig 3, which is a scatter plot showing the relationships between the variables included in the study. From Fig 3, it can be noted that the distribution of agricultural land area indicated heterogeneous variances across all the other variables presented in the scatter plot matrix. The Generalised Least Squares (GLS) is one of the techniques, which is modification of ordinary least squares that considers inequality of variance in the observations. Quantile regression is an alternative method for estimating the functional relationship of the dependent variable and the covariates for all portions of probability distribution [93, 94]. As demonstrated by Mosteller and Tukey [95], it was possible to fit regression curves to other parts of the distribution of the response variable. Applying the standard regression model in such cases where variance is not constant may underestimate, overestimate or become unsuccessful in distinguishing nonzero changes in heterogeneous distributions [96–98].

### 3.4. Model validation

Several model validation analyses were conducted for each of the regression models showing the relationship between the response variable and its determinants as specified in previous sections. These are Mean Error (ME), Root Mean Square Error (RMSE), Mean Absolute Error (MAE), and Relative Difference (RD). The formulas of these measures are given as

$$ME=\frac{\sum_{i=1}^{n}\left(Y_{i}-{\hat{Y}}_{i}\right)}{n}$$ (4)

$$RMSE=\sqrt{\frac{\sum_{i=1}^{n}{\left(Y_{i}-{\hat{Y}}_{i}\right)}^{2}}{n}}$$ (5)

$$MAE=\frac{\sum_{i=1}^{n}\left|Y_{i}-{\hat{Y}}_{i}\right|}{n}$$ (6)

$$RD=\left(\left(\frac{\sum_{i=1}^{n}{\hat{Y}}_{i}}{\sum_{i=1}^{n}Y_{i}}\right)-1\right)\times 100$$ (7)

where Y_i_ is the i^th^ observation of the response variable; ${\hat{Y}}_{i}$ is its predicted value; and n is the number of observations. ME, RMSE, and MAE are the average deviation measures in the study area; and RD represents the difference between estimated and observed values of the response variable by indicating the magnitude and sign of the deviation. The negative values of RD point to underestimation, whereas the positive values refer to overestimation.

**Fig 4:**
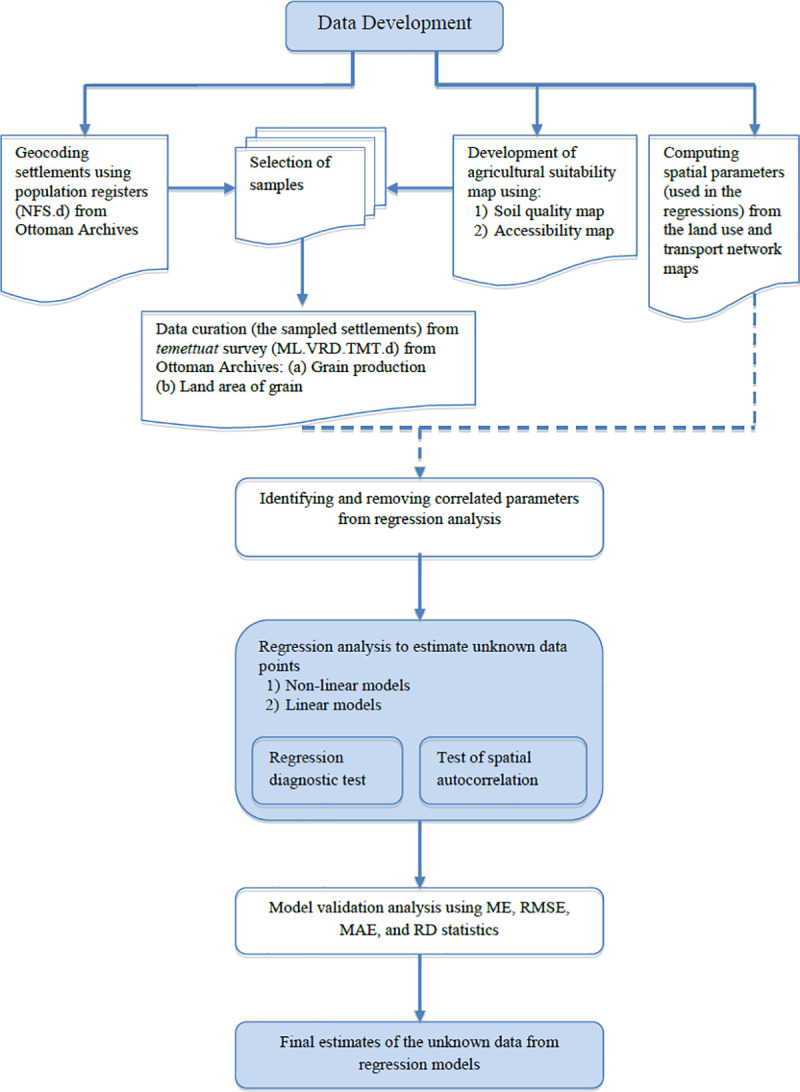
Flowchart for the spatial data development, multiple regression and model validation analysis.

Finally, the steps followed in the methodological process are presented in Fig 4. As shown in the Figure, a sample selection method was first applied using two sources, i.e. geocoding the settlements and development of an agricultural suitability map. Data were curated from the Ottoman Archives regarding the grain production and its cultivation area for the selected samples. In regression analysis, parameters such as elevation, accessibility, distance to water sources, soil quality were computed in ArcGIS using the land use map, transportation map, and soil quality map. The spatial and agricultural production data were analyzed to detect the correlated variables, which were excluded from the regression models. The regression models were estimated using the STATA software. The model validation analysis follows this to select the best performing models. The best models were used for the estimation of data for unknown data points.

## 4. Results from regression analysis

To compare estimations and to evaluate the fitting performance of different models, we have considered NSUR (Models 1), NLS (Model2), TPR (Model3), QREG (Model4), OLS (Model5), and GLS (Model 6) estimators for each agricultural model where the agricultural product or agricultural land area are the response variables. The presented variables in Table 1 were used in the regressions except those indicating a correlation with the other variables (see Table A1 in the S1 Appendix). Table 4 presents the estimated coefficients, which are significant at either 5% or 10% for each of the six different models.

**Table 4:** Results of the global models estimated for the sample of Bursa settlements.

	VALUE OF ESTIMATED PARAMETERS					
	NONLINEAR MODELS		LINEAR MODELS			
	NSUR	NLS	TPR	QREG^1^	OLS	GLS
*Dependent variable*: *AGRI_PRODUCT*	MODEL 1	MODEL2	MODEL3	MODEL4	MODEL 5	MODEL 6
β_AGRI_SUIT_	-	-	-	-	58.32(12.11)**	-
β_LAND_AREA_	0.663(0.04)**	0.595(0.04)**	-	-	0.073(0.03)**	-
β_DIST_SET_CENT_	-	-	-	-	0.072(0.02)**	-
β_ELEVATION_	-0.337(0.06)**	-0.402(0.06)**	-	-	-0.099(0.05)*	-
β_POPULATION_	0.552(0.04)**	0.701(0.05)**	-	-	0.142(0.05)**	-
α_1_	-	-	-	-	-464.15(99.2)**	-
Number of observations	72	72	-	-	72	-
R-square	0.79	0.83	-	-	0.50	-
*Dependent variable*: *AGRI_LAND*						
ρ_AGRI_SUIT_	2.942(0.28)**	-	1.183(0.01)**	26.442(7.82)**	-	53.51(18.08)**
ρ_HH_NUM_	-0.302(0.15)**	-	0.003(0.01)**	0.333(0.19)*	-	-0.465(0.23)**
ρ_ELEVATION_	0.361(0.07)**	-	0.003(0.00)**	-	-	-
ρ_DIST_WATER_	-	-	-	-	-	-0.009(0.00)**
ρ_DIST_ROAD_	-	-	-0.0004(0.00)**			
ρ_DIST_SET_CENT_	-	-	0.001(0.00)**	-	-	0.172(0.02)**
ρ_DIST_RES_CENT_	-	-	-	-0.001(0.00)*	-	-
α_2_	-254.43(98.72)**	-	-3.481(0.14)**	-102.84(53.77)**	-	-345.1(127.9)**
Number of observations	72	-	84	84	-	84
R-square	0.28	-	0.50	0.18	-	-
*Dependent variable*: *PRODUCT_TAX*			-	-	-	-
ρ_AREA_	0.322(0.09)**	-				
ρ_AGRI_SUIT_	3.799(0.32)**	-	-	-	-	-
α_3_	2.784(1.07)**	-	-	-	-	-
Number of observations	72	-	-	-	-	-
R-square	0.34	-	-	-	-	-

**Note:** NSUR: Nonlinear Seemingly Unrelated Regression; NLS: Nonlinear Least Squares Regression; TPR: Truncated Poisson Regression; QREG: Quantile Regression; GLS: Generalised Least Squares Regression.

^t^Quantile (50) *p<0.10 **p<0.05.

In general, the estimates of agricultural land use and production determinants have consistent signs throughout all the models. Soil quality, agricultural land area, and population have positive and significant effects on agricultural production. In some cases, distance to settlement centers has a positive influence, whereas elevation negatively influences agricultural production. This implies that agricultural production is higher in locations that are more distant to settlement centers and lower in higher elevation locations. The reason can be the existence of high-quality agricultural land in the peripheral locations far from settlement centers. This is also the case for the locations of lower elevation.

Similar to agricultural product estimates, soil quality and the number of households have positive impacts on agricultural land development; however, there are also negative estimates of the number of households in Models 1 and 6. This implies that the larger the number of households, the smaller the agricultural land area required for grain production implying that economies of scale could positively impact land use and result in higher yields per household per hectare. In Models 1 and 6, the number of households is negatively; in Models 3 and 4, it is positively related to agricultural land use. Though magnitudes of the household variable’s coefficient are the same in these models, they have differing signs, therefore failing to be robust estimates. Elevation is positively related to agricultural land use in Models 1 and 3. Therefore, agricultural land development was more common in locations of higher elevation in the study area. Distance to main residential centers negatively influences agricultural land development, indicating that agricultural land expansion is more likely in the locations close to main residential centers. Distance to roads also has a negative coefficient indicating that the closer to the road network, the larger the agricultural land development. The tax on agricultural products is positively related to both soil quality and agricultural land area. This implies that agricultural production and resulting agricultural product tax are higher in high soil quality locations and in the locations where agricultural land areas are larger compared to lower soil quality and smaller agricultural land counterparts.

**Fig 5:**
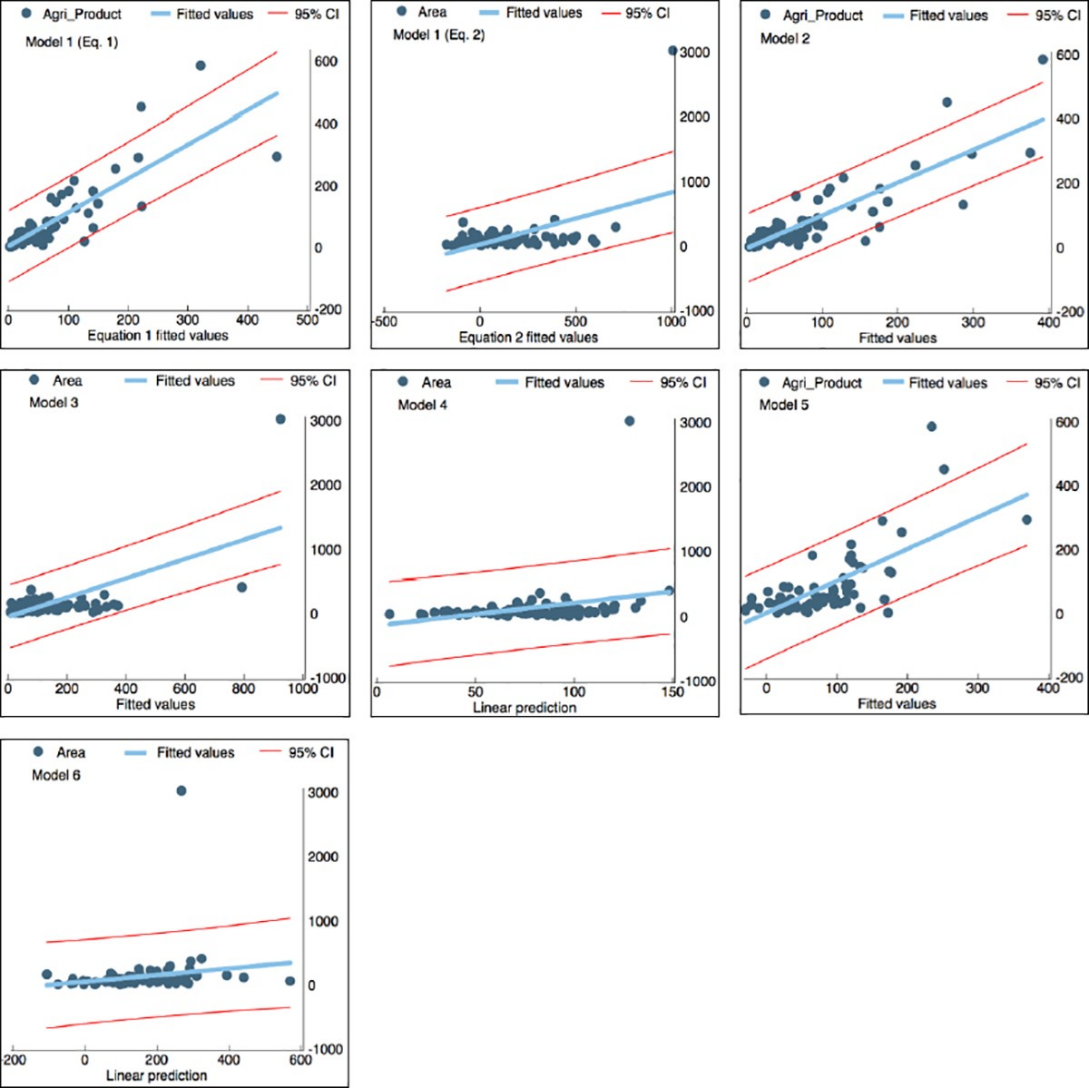
Relationships between observed and fitted values estimated with multivariate regression models for the sub-districts of Bursa.

The scatter plots for the six models showing observed and fitted values are shown in Fig 5. The confidence intervals indicating the reliability of an estimate in the regression are also provided in the Figure where the confidence interval’s width is proportional to the estimator’s standard error. For instance, the larger width of the confidence interval given for Models 1, 5, and 6 indicates that the estimations’ standard error is large, which increases the uncertainty in the estimation of regression coefficients. By contrast, the resulting confidence intervals for Models 2, 3, and 4 are relatively narrow, pointing to a more precise estimation of the regression coefficients.

The R-squared values range between 0.28 and 0.83, indicating that 28% to 83% of the agriculture variables’ variation can be explained by agricultural use determinants as given in Table 3. The deviations from the observed values are the smallest in Model 1 (Eq 2), Model 4, and Model 5 (see Table 5). In Model 4, the RD is 0.01%, while in Model 1 and Model 5, it is 0.38% and -0.54%, respectively. The highest RD is observed in Models 3 and 6, which are -37.9% and %29.4, respectively. These results indicate that Models 2, 4, and 6 overestimate, and Models 1, 3 and 5 underestimate agricultural use’s true values. Throughout the six models, the MAE and ME are the smallest in Model 2 and Model 4, respectively, and are the highest in Models 3 and 6.

**Table 5:** Findings from model validation analysis.

	Model 1 (Eq 1)	Model 1 (Eq 2)	Model 2	Model 3	Model 4	Model 5	Model 6
ME	7.76	-0.49	-3.62	46.21	0.01	0.41	-35.86
RMSE	485.69	2474.5	442.03	2954.23	2031.32	594.01	3001.5
MAE	31.48	154.38	31.80	78.64	97.62	46.64	130.45
RD	-10.30	0.38	4.81	-37.97	0.01	-0.54	29.47

Note: ME: mean error; RMSE: root mean square error; MAE: mean absolute error; RD: relative difference.

Spatial autocorrelation refers to the presence of systematic spatial variation in a variable where the corresponding data values tend to be clustered or dispersed in space. We used Moran’s I index to detect spatial autocorrelation effects concerning the estimated residuals across the specified six models, which we estimated using different regression techniques. Table 6 presents Moran’s I statistics results, which were computed using inverse distance and fixed band methods. From the inverse distance method, Moran’s I ranges between 0.007 and 2.618, indicating no spatial autocorrelation for all the models except Model 1(Eq 2) and Model 6. The resulting p values range between 0.005 and 0.959, indicating that with the p values greater than 0.05 or 0.10, we do not reject the null hypothesis i.e. the spatial variable is randomly distributed. From the fixed band method, the smallest Moran’s Index was 0.002, and the biggest value was 2.282. The p values range between 0.004 and 0.555, where we reject the null hypothesis for Models 1(Eq 2) and 6, indicating that there is spatial autocorrelation observed in these models. For the rest of the models, spatial autocorrelation is not a significant issue confirming no model specification errors regarding the subject models.

**Table 6:** Results from spatial autocorrelation statistics.

	Model 1 (Eq 1)	Model 1 (Eq 2)	Model 2	Model 3	Model 4	Model 5	Model 6
Moren’s Index (1)	0.111	2.191	0.017	0.014	0.035	0.007	2.618
Z-score	0.141	2.388	0.032	0.159	0.050	0.021	2.803
p-value	0.887	0.016	0.974	0.872	0.959	0.983	0.005
Moren’s Index (2)	0.017	2.282	0.068	0.002	0.063	0.061	0.103
Z-score	0.881	2.377	2.011	0.590	1.840	1.815	2.820
p-value	0.378	0.017	0.050	0.555	0.070	0.069	0.004

Note: Moren’s Index (1) were calculated using the inverse distance method; Moren’s Index (2) were calculated using the fix band method.

**Fig 6:**
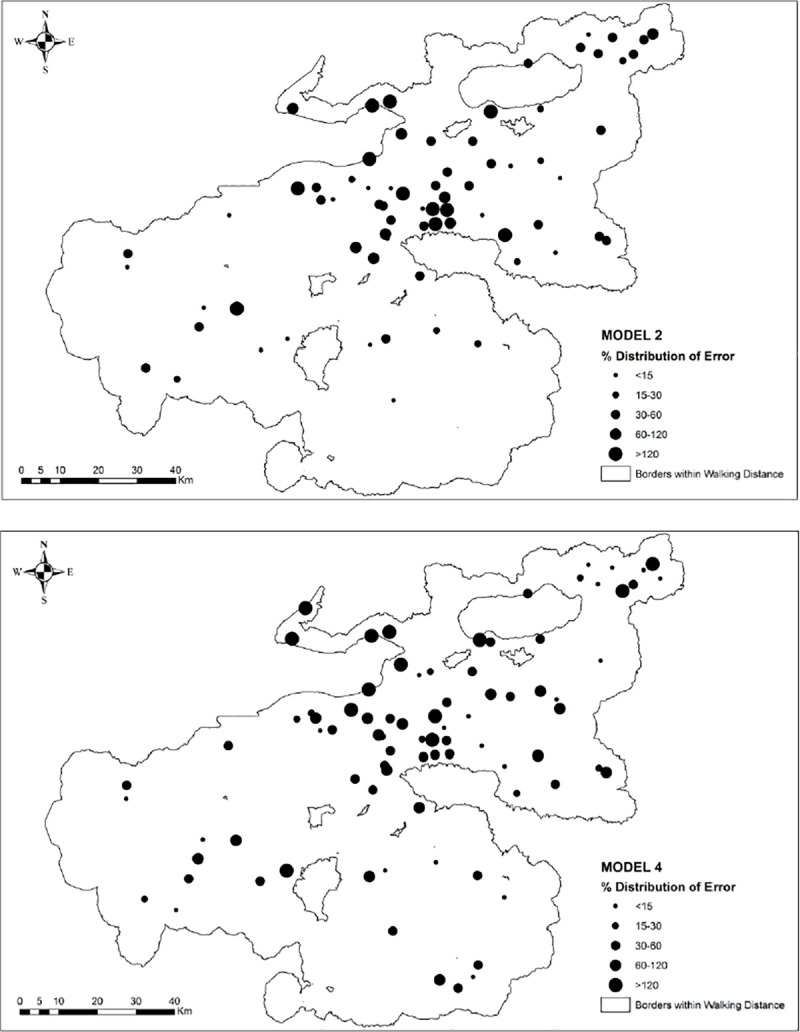
Deviation of predicted values from observed values across different models estimated for Bursa settlements (a) Model 2 (b) Model 4.

**Fig 7:**
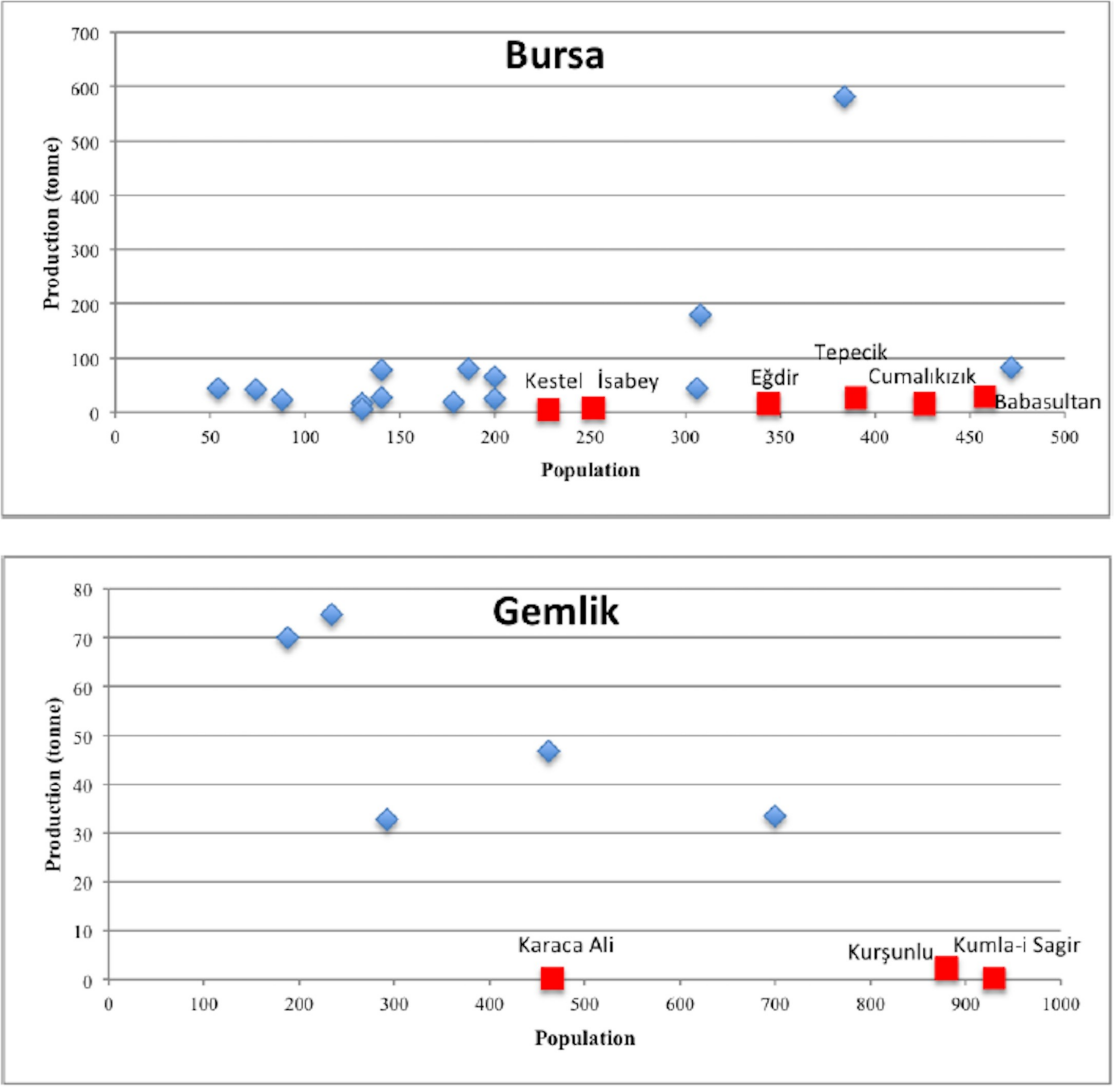
The observed relationship between population and agricultural production in Bursa and Gemlik.

Based on the results from model validation analysis (Table 5) and spatial autocorrelation statistics (Table 6), we found that Model 2 and Model 4 perform better than other models. Therefore we considered Model 2 for the estimation of agricultural production and Model 4 to estimate its cultivation area. The spatial distribution of variations of predictions from the observed values of agricultural response variables from Models 2 and 4 is provided in Fig 6. We note that the models, in general, overestimated the agricultural variables with the deviations from the true value of the response variable ranging between 1% to more than %120. There are spatial variations with the distribution of errors in both models. The largest estimation errors were observed in İznik, Gemlik, and Bursa, where the other sub-districts resulted in relatively smaller errors. Regarding production estimates (Fig 6A), higher errors are observed for the settlements in İznik, Kite, Gemlik, Mudanya, Mihaliç, and Bursa. In contrast, lower errors are reported for Kirmasti, Atranos, Mihaliç, and İnegöl. The subject settlements having the highest estimation errors have considerably higher or lower agricultural production values compared to the mean values of production computed for each sub-district. For instance, Karaca Ali, Kumla-i Sagir, Kurşunlu, Kestel, Cumalıkızık, and Sölöz were reported for having a considerably lower amount of grain production ranging from 0.2 to 17.3 tonnes compared to grain production of other settlements of Bursa Region. Among these, Sölöz was specialized in silk production, whereas Kumla-i Sagir and Kurşunlu were specialized in olive and wine productions. Therefore, these locations that were identified as outliers can be characterized as local concentrations of agro-industrial production. In other words, these were exporting agro-industrial products, as they were dependent on other settlements for grain demand. When we examine the relationship between population and agricultural production (Fig 7), we note that the settlements associated with the highest estimation errors have a relatively high population and considerably low production values. Examples from these settlements that are marked with red color are shown in Fig 7.

**Fig 8:**
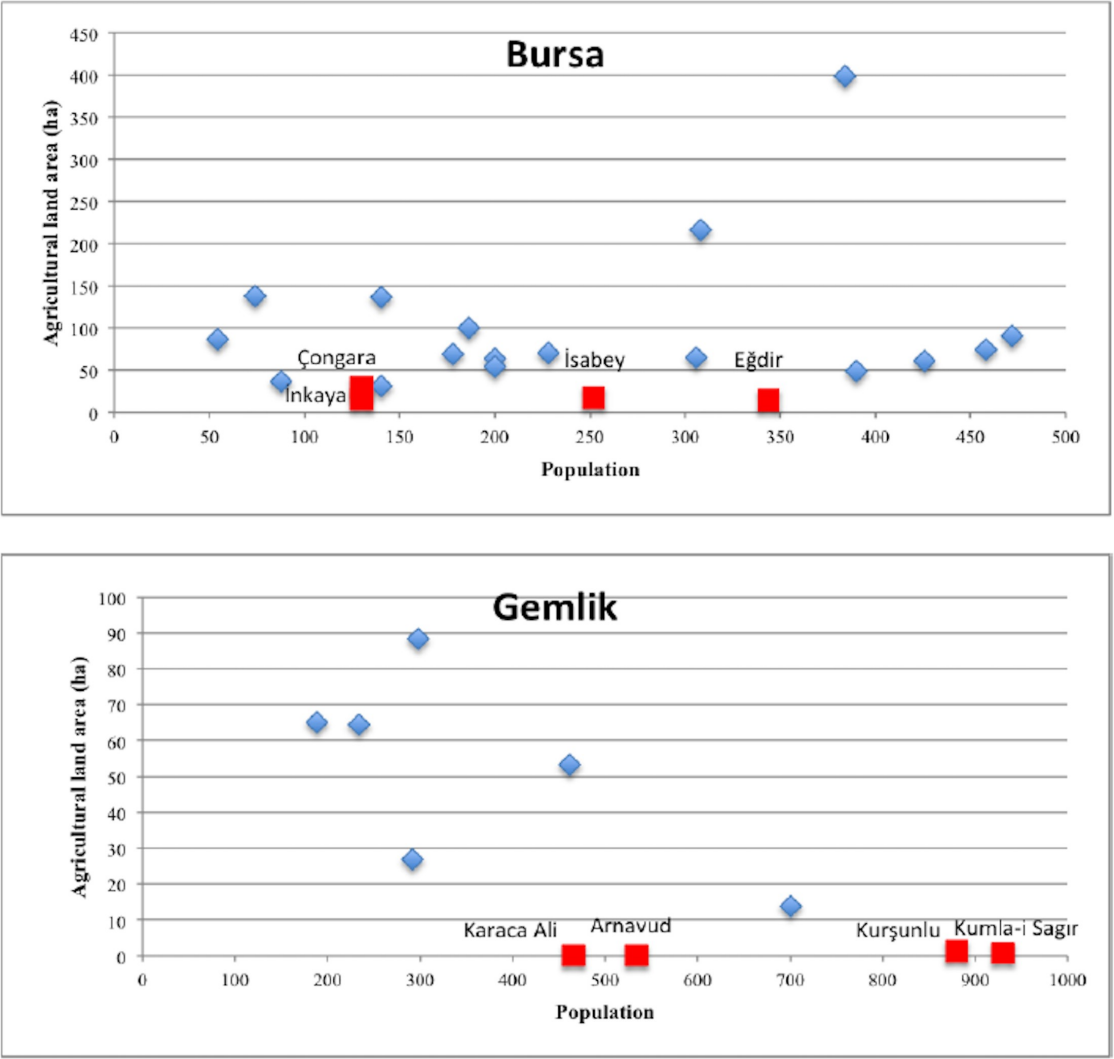
The observed relationship between population and agricultural land area in Bursa and Gemlik.

Regarding agricultural land estimations (Fig 6B), Bursa, Gemlik, İznik, Mihaliç, and Mudanya are the sub-districts that are associated with the highest estimation errors. Similar to agricultural production estimates, the highest estimation errors were observed for the settlements with a high population and a considerably small agricultural land. These settlements are among those located in the coastal area having a relatively lower amount of land for grain production than other settlements in inner locations. Due to physical limitations, it is possible that these settlements were satisfying their grain demand from other locations which had excess grain production. Some examples are shown in Fig 8.

**Fig 9:**
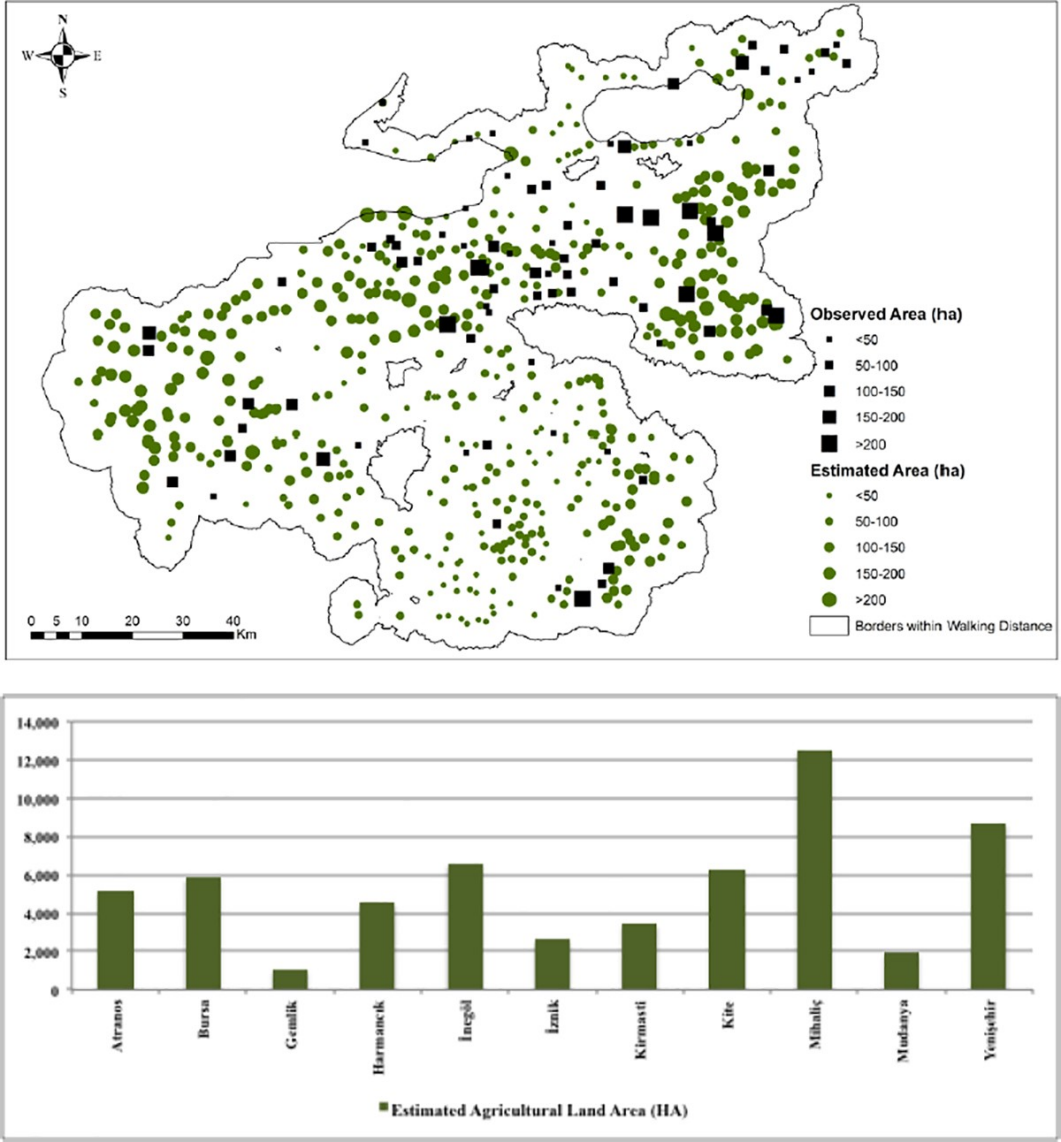
Model 4 findings on predicted and observed values of the agricultural land area (ha) in Bursa settlements.

We used the estimated relationships between the response and explanatory variables from Model 2 and Model 4 to estimate the missing data points’ agricultural information. The observed and estimated agricultural land area values from Model 4 are presented in Fig 9, and some statistics on the estimated values concerning 11 sub-districts (see Fig 2) are provided in Table 7.

**Table 7:** Summary statistics of estimated and observed agricultural land area (in ha) from Model 4.

Sub-district	Number of settlements	Sum	Mean	SD	Min	Max
Atranos	108	5124	47.40	12.28	6.39	78.57
Bursa	66	5849	88.62	52.37	14.43	398.14
Gemlik	19	1011	53.22	56.61	0.18	247.44
Harmancık	43	4513	104.95	22.38	38.79	200.15
İnegöl	44	6590	149.77	62.09	28.50	357.76
İznik	36	2621	72.79	34.87	15.24	169.08
Kirmasti	34	3430	100.87	40.94	34.77	271.46
Kite	61	6258	102.58	48.82	21.42	289.63
Mihaliç	103	12485	121.21	59.98	17.27	610.94
Mudanya	21	1900	90.48	58.79	31.43	252.20
Yenişehir	41	8697	212.13	451.34	41.76	3015.57

**Note:** City of Bursa was not included in the analysis due to its considerably large population size.

According to Model 4 estimations, Mihaliç, Yenişehir, İnegöl, and Bursa are the top sub-districts having the largest agricultural land area in the Region. These settlements are characterized by having the most fertile land in the Region. In particular, Mihaliç and Bursa are among the highly populated sub-districts in Bursa Region. By contrast, Gemlik and Mudanya are the sub-districts having the smallest agricultural land area. As summarised in Table 7, Atranos, Mihaliç, Bursa, and Kite have the highest number of settlements, with Gemlik and Mudanya having the smallest number. The min. and max. values of land area are observed for Gemlik and Yenişehir, respectively. Regarding the mean values of the agricultural land area, Atranos and Gemlik have the lowest, and Yenişehir and İnegöl have the highest mean values.

**Fig 10:**
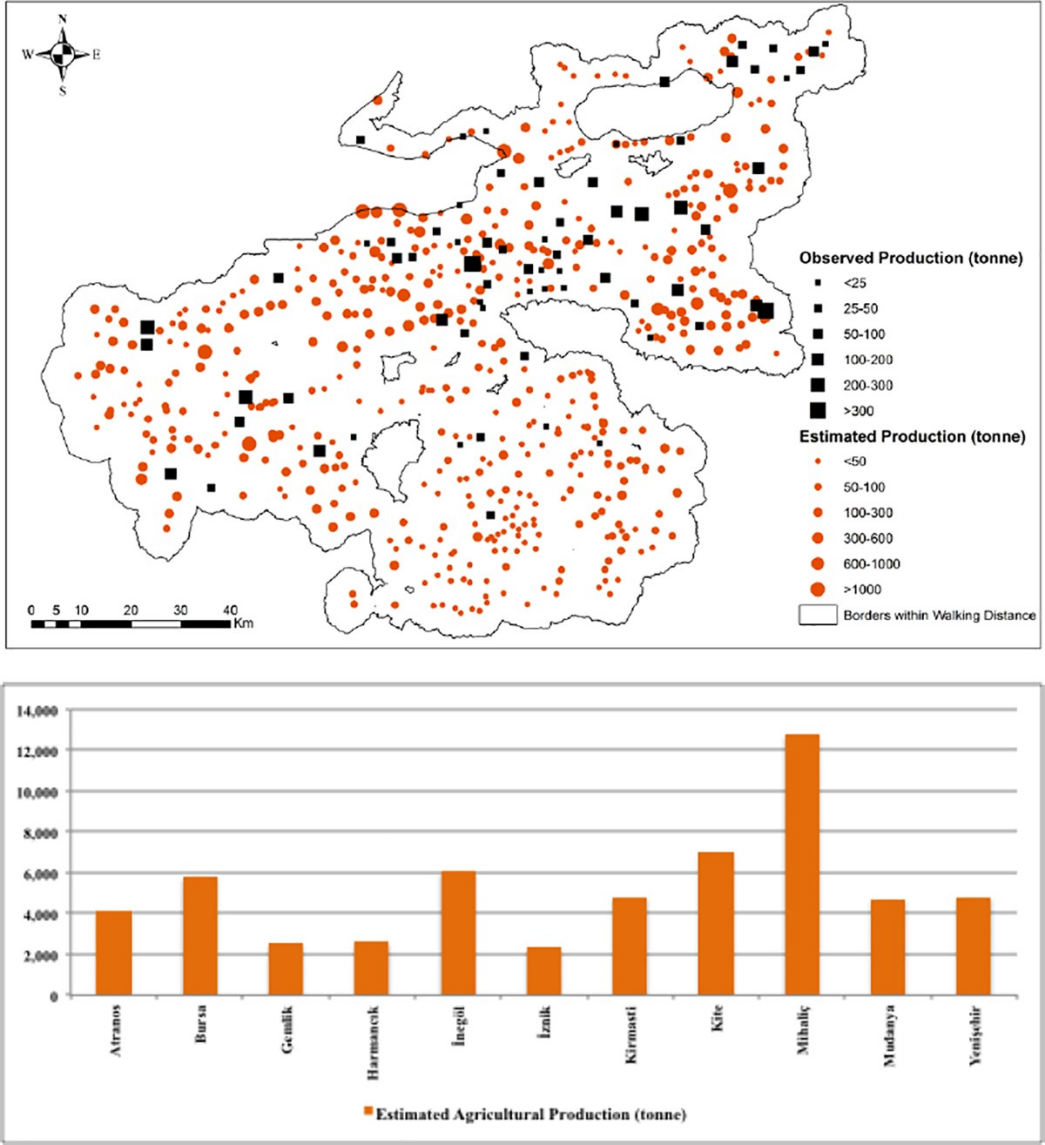
Model 3 findings on predicted and observed values of the agricultural production (tonne) in Bursa settlements.

**Fig 11:**
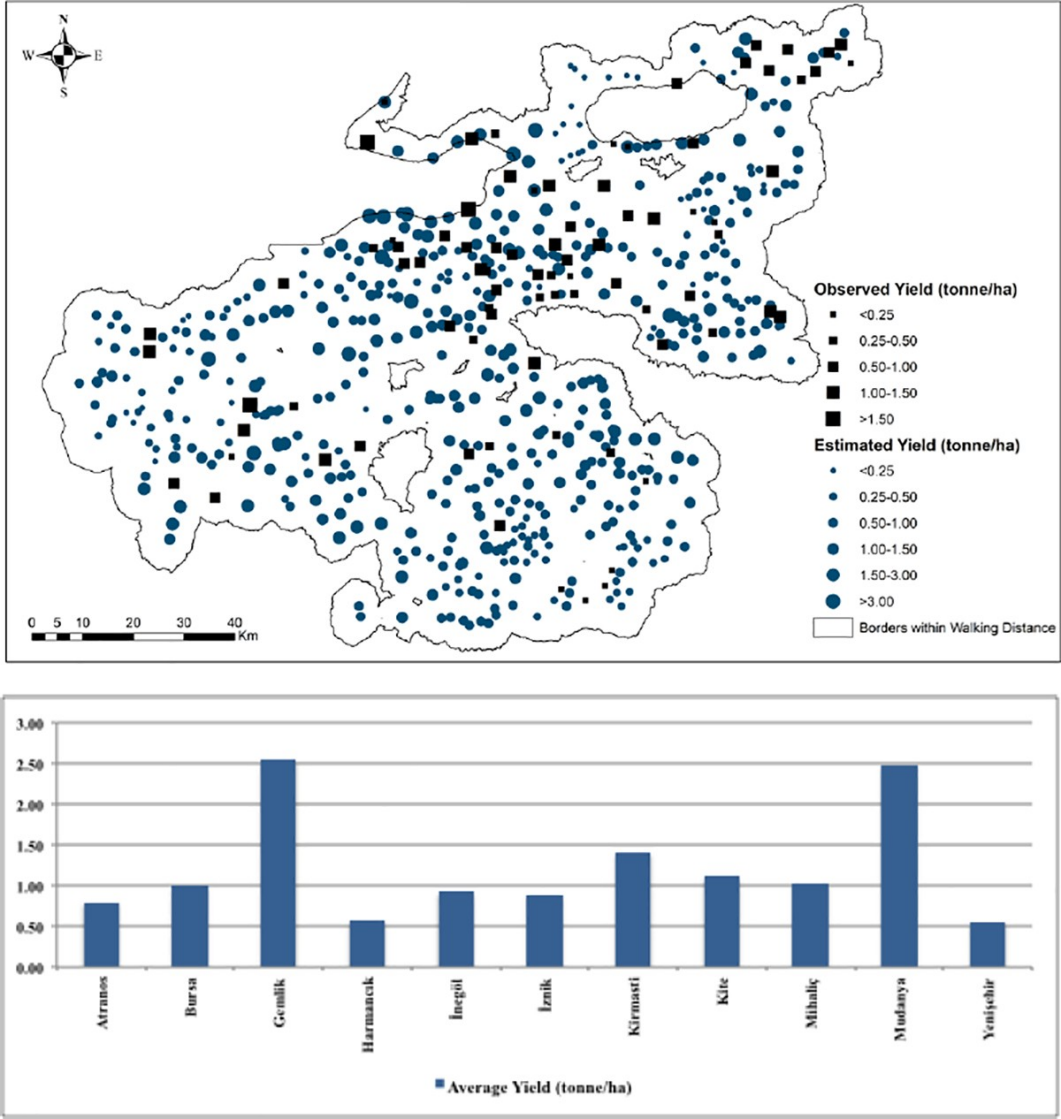
Distribution of average yield (tonne/ha) of predicted and observed values.

Fig 10 presents the observed and estimated values of non-irrigated production of grains given in tonnes. The largest amount of grain production was estimated for Mihaliç, Kite, Bursa, İnegöl, and Yenişehir while the lowest figures are observed for Harmancık, Gemlik, and İznik. From the summary statistics for the estimated values of agricultural production (Table 8), the min. value of production was observed for Gemlik and the max. value was for Mihaliç. The mean values of agricultural production indicated that Atranos has the lowest and Mudanya has the highest mean production values. We also computed average yield values that we obtained from regression estimates, which are presented in Fig 11. It can be seen from the Fig that Gemlik, Mudanya, and Kirmasti produced the highest yields of non-irrigated crops which are followed by Bursa, İnegöl, Mihaliç, and İznik. Harmancık and Yenişehir are the two sub-districts associated with the lowest grain yields.

**Table 8:** Summary statistics of estimated and observed agricultural production (in tonne) from Model 2.

Sub-district	Number of settlements	Sum	Mean	SD	Min	Max
Atranos	108	4093	37.89	24.58	5.39	137.18
Bursa	66	5792	87.75	108.81	5.26	582.26
Gemlik	19	2572	135.38	258.9	0.26	1131.6
Harmancık	43	2601	60.48	25.12	24.7	160.47
İnegöl	44	6063	137.79	171.53	15.27	911.12
İznik	36	2340	65.01	58.58	10.13	334.03
Kirmasti	34	4820	141.76	298.31	14.34	1797.53
Kite	61	6982	114.45	139.21	15.21	920.24
Mihaliç	103	12781	124.09	355.72	13.13	3646.01
Mudanya	21	4703	233.94	371.27	22.83	1322.6
Yenişehir	41	4824	117.65	186.08	12.18	1220.44

**Note:** City of Bursa was not included in the analysis due to its considerably large population size.

We analyzed the relationship between population and estimated production values for all the 11 sub-districts included in the study for confirmation purposes. A statistically significant positive relationship was uncovered between population and estimated values for each of the 11 sub-districts in the Bursa Region (some examples are presented in Fig A1 in the S1 Appendix). These findings align with the expectations suggesting that an increase in population in the Bursa Region sub-districts resulted in a parallel increase in grain production in the corresponding sub-district. Similarly, the same relationship between population and agricultural land area was searched for the selected regression model (Model 4). Its positive relationship with population confirms our expectation that there was a land expansion process associated with the population growth in the sub-districts of Bursa.

Finally, we provide the data on estimated values of land area, production, and yield for the Bursa Region’s selected settlements in Table 9. The information on the remaining settlements can be obtained from the authors on request.

**Table 9:** Details of estimates of agricultural land area, production and yield in selected settlements of Bursa Region.

Sub-district	Settlement Name	Land Area (ha)	Production (tonne)	Yield (tonne/ha)
Atranos	Ağaçhisar	47.88	50.53	1.06
	Bayındır	37.12	17.09	0.46
	Çeribaşı	61.82	33.42	0.54
	Delice	43.89	34.31	0.78
	Güney	39.89	48.43	1.21
	Haydar	44.83	46.32	1.03
	Karıncalı	44.67	51.54	1.15
	Orta	39.60	31.67	0.80
	Sorgun	49.62	82.44	1.66
	Yenice	60.41	41.11	0.68
Bursa	Avdancık	81.49	110.72	1.36
	Canbazlar	105.83	80.23	0.76
	Demirtaş	194.49	550.92	2.83
	Dimboz	58.63	45.77	0.78
	Gölbaşı	68.96	45.24	0.66
	Hasan	102.14	84.30	0.83
	Karaman	86.25	25.76	0.30
	Panayır	90.04	32.62	0.36
	Seç	87.55	170.24	1.94
Gemlik	Armutlu	14.03	33.55	2.39
	Benli	83.90	284.22	3.39
	Ericek	16.76	16.30	0.97
	Fıstıklı	36.39	51.06	1.40
	Gençali	66.96	99.50	1.49
	Kapaklı	53.35	70.11	1.31
	Kumla-i Kebir	27.43	52.71	1.92
	Narlı	21.13	35.93	1.70
	Nefs-i Gemlik	247.44	1131.63	4.57
	Umurbey	121.82	355.75	2.92
Harmancık	Avdan	94.67	43.97	0.46
	Dedebali	91.53	54.18	0.59
	Eşen	107.52	60.16	0.56
	Hereke	90.33	63.86	0.71
	Karaca	107.86	42.74	0.40
	Kılaguzlar	111.27	25.62	0.23
	Kozluca	98.25	31.94	0.33
	Nusratlar	92.67	27.04	0.29
	Sırıl	88.32	43.23	0.49
	Yunuslar	102.50	53.24	0.52
İnegöl	Alibey	171.63	149.41	0.87
	Bedre	111.25	112.94	1.02
	Çitli	159.26	171.82	1.08
	Eymir	136.91	75.09	0.55
	Hoca	155.35	110.26	0.71
	Kızık	81.89	15.45	0.19
	Maden	114.86	114.60	1.00
	Orta	155.90	114.51	0.73
	Sırnaz	123.97	90.45	0.73
	Tukaş	133.76	19.46	0.15
İznik	Belheriz	64.55	44.98	0.70
	Çakırca	109.54	106.98	0.98
	Dere	76.17	51.34	0.67
	Epsere	55.84	13.96	0.25
	Hoca	78.58	32.17	0.41
	İnikli	72.16	54.52	0.76
	Maraga	80.02	19.12	0.24
	Narlıca	65.62	73.42	1.12
	Ömerli	92.72	121.45	1.31
	Tacir	65.64	106.99	1.63
Kirmasti	Behram	74.03	84.45	1.14
	Demirili	83.76	110.17	1.32
	Gerede	114.61	34.57	0.30
	Kadı	103.71	162.59	1.57
	Kayıkçı	45.12	58.98	1.31
	Mudam	90.15	114.02	1.26
	Sarıbey	135.51	82.50	0.61
	Üçbeyli	81.17	110.14	1.36
	Yamanlı	113.41	50.39	0.44
	Yumucaklı	105.45	78.45	0.74
Kite	Balıklı	122.27	79.67	0.65
	Demirci	142.85	183.47	1.28
	Erenler	57.70	72.50	1.26
	Fodra	148.07	154.52	1.04
	Gököz	60.32	36.01	0.60
	Hasanağa	153.93	227.18	1.48
	Kirazlı	69.73	114.24	1.64
	Nalınlar	56.00	67.97	1.21
	Tuzaklı	51.11	56.37	1.10
	Yaylacık	137.82	183.65	1.33
Mihaliç	Bulgar	166.14	64.64	0.39
	Çakıl	132.04	124.62	0.94
	Delice	59.23	88.66	1.50
	Esemen	156.96	55.00	0.35
	İkizce	130.57	177.90	1.36
	Karacalar	87.02	67.29	0.77
	Melik	108.68	145.62	1.34
	Onaç	53.34	47.04	0.88
	Tophisar	159.83	75.93	0.48
	Yeni	129.62	47.69	0.37
Mudanya	Altuntaş	41.31	47.87	1.16
	Bacala	74.30	61.92	0.83
	Çekrice	78.55	50.84	0.65
	Dere	108.26	363.11	3.35
	Frenkli	53.07	63.52	1.20
	Kızıl	55.98	71.46	1.28
	Misebolu	115.58	304.92	2.64
	Mürsel	92.71	90.32	0.97
	Yenice-i Müslim	88.52	42.27	0.48
	Yörüklü	72.88	81.34	1.12
Yenişehir	Afşar	133.50	41.60	0.31
	Burcun	88.40	80.13	0.91
	Ebe	133.80	98.69	0.74
	Kızıl	95.68	100.84	1.05
	Makri	177.78	198.15	1.11
	Okuf	135.04	78.75	0.58
	Rüstem	130.25	94.82	0.73
	Subaşı	152.41	130.72	0.86
	Terzi	128.52	142.80	1.11
	Ulu	148.12	15.83	0.11

## 5. Discussion

Our results show that agricultural production and its cultivation area increase relative to soil quality, agricultural land area, household number, and population. This is in line with the previous research findings, which asserted that agricultural production is positively correlated with soil quality [99, 100], cultivation area [101, 102], and population [103, 104]. We found that grain production is positively related to distance to settlement centers indicating that locations farther from settlement centers produced more grain than closer locations. Similar to this finding, Bastian et al. [105] found that the more distant and rural the farmland, the higher the land price. This implies that peripheral locations had more rural character and might have higher quality and higher value land than inner locations.

According to the classical representation of land use pattern early in the nineteenth century (Von Thunen, [106]), market processes determine the use of a particular piece of land where economic rent is the principle factor. According to Von Thunen’s model, transport costs were the main factor determining economic rent; hence the highest bid for the land and displace all others. According to our findings, distance to roads and distance to residential centres relate negatively to the agricultural land area indicating that transport costs cause the economic rent to be diminished for each unit of distance; hence confirming Von Thunen’s theory. Von Thunen stated that agricultural commodities which yield a lower bulk per hectare (e.g. grain) do not yield a higher rent close to the market compared to commodities which yield a large bulk per hectare (e.g. potatoes). Because costs of transportation of grain per hectare are relatively lower and its value per unit of weight is relatively higher, economic rent diminishes slowly with distance from the market. The positive coefficient on distance to settlement centres estimated for grain production and its cultivation area in Bursa Region supports this statement given that in more distant locations from the settlement centres, economic rent is high enough to support grain production. Von Thunen’s theory was applicable in the nineteenth century of historical settlements as well as today’s nonindustrialized settlements, which is also verified in our case study where influence of transport costs on agricultural use was the determining factor of crop production and its cultivation area in the Bursa Region in 1840s. Von Thunen’s model can be applicable to Bursa Region given that (i) many of the settlements are self-sufficient in grain production, (ii) there are no climate differences (but there may be differences in soil quality in each settlement), (iii) there is no developed transport system as horse- or ox-drawn carts were the only vehicles used in the Region, and (iv) except the settlements located in the plains of the mountain, there is a uniform plain in the Region.

In Von Thunen’s model, the most inner zone (around central core of residential land) is occupied by perishable products (e.g. vegetables and fresh milk); which is followed by woodland as both have high transportation costs. The following three zones are crop farming zones of gradually decreasing intensity. There is evidence in the literature that there are settlements accorded closely to Von Thunen’s principles both in historical periods and in underdeveloped regions in modern times. For instance, Ewald [107] studied Indian and Spanish economies in the colonial period and revealed that distinct rings of crop production existed surrounding the built up areas. Other examples are Blaikie [108] and Müller [109] (references are from: O’Kelly and Bryan [110]). In our case study, the existing cadastral maps for the seven rural settlements of Bursa present a similar structure but agricultural land uses are more heterogeneously distributed by contrast to the theoretically homogeneous zones of Von Thunen’s model. For instance, in some settlements woodland is located in the most inner zone which are in dispersed patches, and this is followed by vegetable gardens which are scattered in the area. The reverse may apply in some other settlements coinciding with the Von Thunen’s model. We cannot reach to a general conclusion that Von Thunen’s model provides an adequate explanation of the spatial structure of land use in Bursa Region in 1840s given that we do not have the historical cadastral maps of land cover/use for all the settlements in the Region. However, the estimations of cropland from this study can be used in line with the Von Thunen’s model for spatial reconstruction of rural land in the Bursa Region in future studies. For such a theoretical reconstruction, the knowledge on land area of other agricultural uses (e.g. vegetable gardens, cattle grazing, groves, etc.) is essential. This data can be curated from the documents obtained from Ottoman Archives.

From our regression models, the agricultural land area was positively related to elevation while grain production was related negatively. This indicates that at higher elevations, agricultural land area is increasing while production is decreasing. Jiang et al. [111] found that intensity of agricultural use is negatively related to elevation, implying a land extension rather than intensification at higher elevations, which complies with our findings. Similar to Jiang et al. [111] and Volante et al. [112], we found that expansion of agricultural land is more likely in the locations that are close to main residential centers. The reason can be the ease of marketing the agricultural products to the main agricultural markets located nearby. Due to accessibility issues, being close to the road network was important for agricultural land development. This verifies the findings of Qin and Zhang [113], which stated that access to roads improves specialization in agricultural production, and with better road connection, household agricultural income increases with a poverty reduction (see also Volante et al. [112]; Gasparri et al. [114]). Finally, distance to watercourses was negatively correlated with agricultural land expansion showing the benefits of being close to water sources. This was also emphasized in Das et al. [115] and Assouline et al. [116].

We had inconsistency in the results regarding some variables such as elevation and household number that we estimated for grain production and agricultural land area. It is also important to note that the factors influencing grain production and its cultivation area are different across the models that we estimated. For instance, Model 2 indicates that elevation, population and land area are the factors explaining grain production. According to Model 4 findings, soil quality, number of households and distance to main residential centers are the significant determinants of grain cultivation area. The reason for these inconsistencies is that the data entries for the grain production do not match with those of the cultivation area. This raises the issue of data quality as we had to rely on the only available data where grain production data entries do not match with its cultivation area values. As we noted previously, this is the only data that is available at the greatest detail for the nineteenth century of the Bursa Region and it is impossible to have higher quality data in our case.

## 6. Conclusion

In this study, we estimated the total area assigned to, and total volume of production of, non-irrigated crops, as the closest proxy for grain cultivation, for Bursa Region for all 576 (except the city of Bursa) settlements in the 1840s by using regression models based upon a geo-sampled total of 72 settlements. We used different nonlinear and linear models for the estimation of agricultural uses. By doing this, it was possible to select the best fitting models through the model evaluation criteria. Considering the issues of nonlinearity in agricultural production and the heterogeneous variances in agricultural land area data, we selected two specific models that proved to be effective in dealing with nonlinearity and heterogeneity issues. These models provided more accurate estimations and were better in considering sub-regional dynamics within our chosen area. However, our selected models have underestimated or overestimated the agricultural use variable, particularly for the regions associated with very low grain production levels or small cultivation areas, both of which correspond to highly populated settlements. For these settlements, our models significantly overestimated agricultural production and cultivation area.

Our results, especially for the sub-districts of Gemlik and Mudanya with their administrative and economic centers on the shore, hints at the overlooked importance of grain trade which has not been explored in detail in historical literature for the 1840s. Similarly, we think we should model the economic and agricultural interplay of the city of Bursa with the remaining settlements in the region in our future studies. Further to this, the modeling already curated data on different agricultural land uses (e.g. permanent crops, permanently irrigated land, pastures, forest, and seminatural areas). In the future, this work will enable us to use the information that we developed in this study and combine it with the other agricultural land use data to reconstruct the spatially-explicit agricultural area and agricultural land cover for the Bursa Region in the 1840s. The historical reconstruction of agricultural land in Bursa will provide a base for future studies through offering possibilities to use these results alongside other scientific research conducted at regional or more local level in Bursa.

With this study, we have developed a method to systematically use an underutilized historical source on agricultural production for our selected region and estimated crop yields for grains for the 1840s. Our approach has two major advantages for future studies on Ottoman agriculture. First, it is scalable as it is based upon sampled observations. Using the same methodology, when sample data is collected, the total volume of grain production and area of cultivation can also be estimated for the entire extent of the survey we used. The survey covers a massive territory in Southeast Europe and Anatolia, which includes today’s Bulgaria, Northern Macedonia, regions of Northern Greece and Southern Serbia, and the Western half of Turkey. By adding new regions following the same methodology, land productivity for grain production can be estimated for several regions with sub-units and inter- as well as intra-regional comparisons can be made for the 1840s Ottoman land productivity. Secondly and more relevant for the Bursa Region since also the population data for all 576 settlements in the region are available, as a second step labor productivity in grain production can also be estimated for rural settlements.

## Supporting information

S1 Data (ZIP)

S1 Appendix (DOCX)
