# Supplementary material for: The estimation of non-irrigated crop area and production using the regression analysis approach: A case study of Bursa Region (Turkey) in the mid-nineteenth century
Source: PLoS One. 2021 Apr 30;16(4):e0251091. doi: 10.1371/journal.pone.0251091 (PMC8087084; doi:10.1371/journal.pone.0251091)
Supplement: S1 Appendix — (DOCX) [file pone.0251091.s002.docx]

**S1 Appendix**

**Fig A1. The relationship between population and estimated production of non-irrigated crops for the selected sub-districts of Bursa Region, 1840s.**


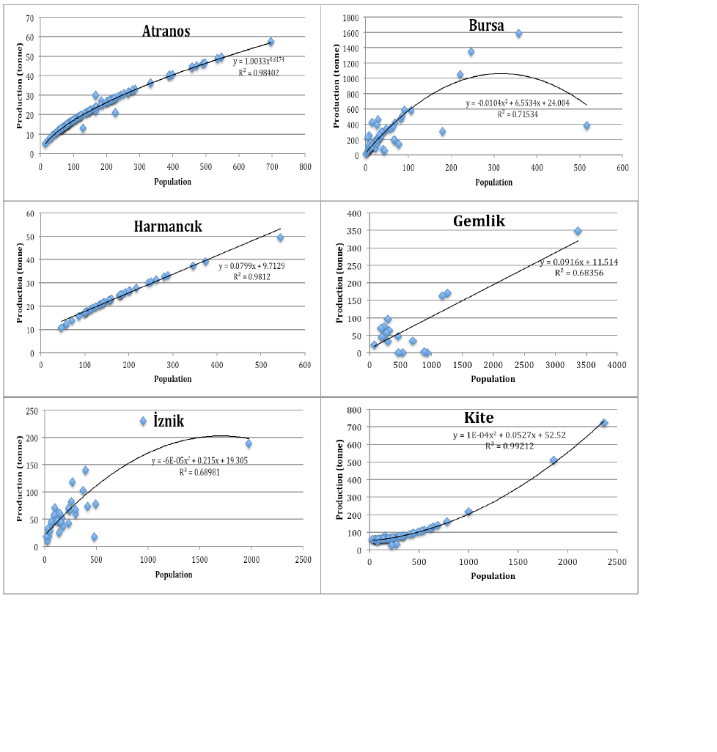


**Fig A2. The relationship between population and estimated cultivated land of non-irrigated crops for the selected sub-districts of Bursa Region, 1840s.**


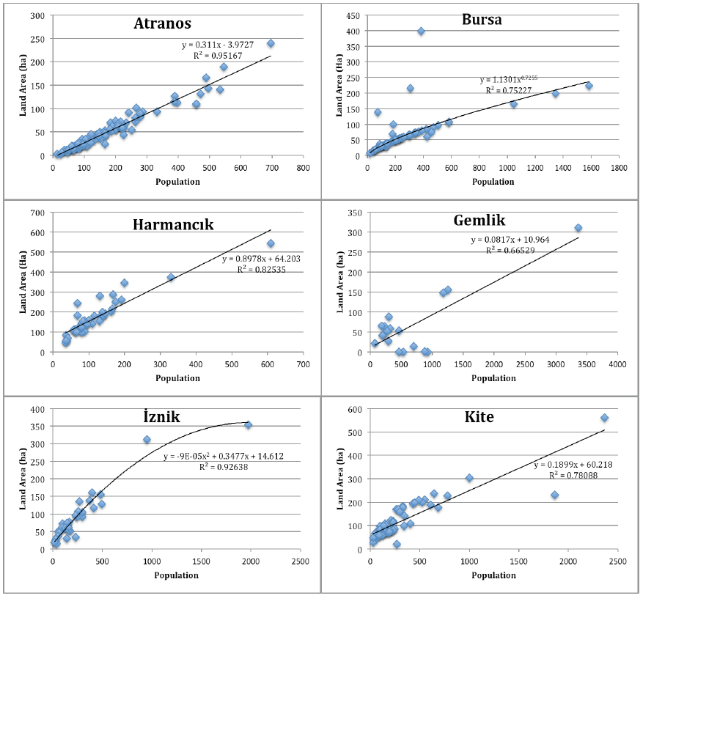


**Table A1. Correlation matrix for the explanatory variable.**

|  | DIST_ROAD | DIST_RES_CENT | DIST_SET_CENT | ELEVATION | DIST_WATER | AGRI_SUIT_INDEX | POPULATION | HH_NUM | PRODUCT_TAX |
| --- | --- | --- | --- | --- | --- | --- | --- | --- | --- |
| DIST_ROAD | 1 |  |  |  |  |  |  |  |  |
| DIST_RES_CENT | 0.426* | 1 |  |  |  |  |  |  |  |
| DIST_SET_CENT | 0.365* | 0.314* | 1 |  |  |  |  |  |  |
| ELEVATION | 0.386* | 0.525* | 0.166 | 1 |  |  |  |  |  |
| DIST_WATER | 0.366* | 0.323* | 0.407* | 0.207 | 1 |  |  |  |  |
| AGRI_SUIT_INDEX | -0.574* | -0.405* | -0.372 | -0.709* | -0.377* | 1 |  |  |  |
| POPULATION | -0.043 | -0.194 | 0.213 | -0.029 | 0.364* | -0.163 | 1 |  |  |
| HH_NUM | -0.068 | -0.251* | 0.233 | -0.05 | 0.363* | -0.179 | 0.968* | 1 |  |
| PRODUCT_TAX | -0.277* | -0.039 | 0.212 | -0.222 | -0.051 | 0.429* | 0.240* | 0.127 | 1 |
